# A Comparison of Double Poling Physiology and Kinematics Between Long-Distance and All-Round Cross-Country Skiers

**DOI:** 10.3389/fspor.2022.849731

**Published:** 2022-04-12

**Authors:** Per-Øyvind Torvik, Øyvind Sandbakk, Roland van den Tillaar, Rune Kjøsen Talsnes, Jørgen Danielsen

**Affiliations:** ^1^Department of Sports Science and Physical Education, Nord University, Meråker, Norway; ^2^Centre for Elite Sports Research, Department of Neuromedicine and Movement Science, Norwegian University of Science and Technology, Trondheim, Norway; ^3^Meråker High School, Trøndelag County Council, Steinkjer, Norway

**Keywords:** electromyography, gross efficiency, kinematics, maximal oxygen uptake, XC skiing

## Abstract

**Purpose:**

The objective of this study was to compare physiological and kinematic responses to double poling (DP) between long-distance (LDS) and all-round (ARS) cross-country skiers.

**Methods:**

A number of five world-class LDS (28.8 ± 5.1 years, maximal oxygen uptake (VO_2max_): 70.4 ± 2.9 ml·kg^−1^·min^−1^) and seven ARS (22.3 ± 2.8 years, VO_2max_: 69.1 ± 4.2 ml·kg^−1^·min^−1^) athletes having similar training volumes and VO_2max_ performed three identical tests; (1) submaximal and incremental tests to exhaustion while treadmill DP to determine gross efficiency (GE), peak oxygen uptake (DP-VO_2peak_), and peak speed; (2) submaximal and incremental running tests to exhaustion to determine GE, VO_2max_ (RUN-VO_2max_), and peak speed; and (3) an upper-body pull-down exercise to determine one repetition maximum (1RM) and peak power. Physiological responses were determined during both DP and running, together with the assessments of kinematic responses and electromyography (EMG) of selected muscles during DP.

**Results:**

Compared to ARS, LDS reached higher peak speed (22.1 ± 1.0 vs. 20.7 ± 0.9 km·h^−1^, *p* = 0.030), DP-VO_2peak_ (68.3 ± 2.1 vs. 65.1 ± 2.7 ml·kg^−1^·min^−1^, *p* = 0.050), and DP-VO_2peak_/RUN-VO_2max_ ratio (97 vs. 94%, *p* = 0.075) during incremental DP to exhaustion, as well as higher GE (17.2 vs. 15.9%, *p* = 0.029) during submaximal DP. There were no significant differences in cycle length or cycle rate between the groups during submaximal DP, although LDS displayed longer relative poling times (~2.4% points) at most speeds compared to ARS (*p* = 0.015). However, group × speed interaction effects (*p* < 0.05) were found for pole angle and vertical fluctuation of body center of mass, with LDS maintaining a more upright body position and more vertical pole angles at touchdown and lift-off at faster speeds. ARS displayed slightly higher normalized EMG amplitude than LDS in the muscles rectus abdominis (*p* = 0.074) and biceps femoris (*p* = 0.027). LDS performed slightly better on 1RM upper-body strength (122 vs. 114 kg, *p* = 0.198), with no group differences in power in the pull-down exercise.

**Conclusions:**

The combination of better DP-specific aerobic energy delivery capacity, efficiency, and technical solutions seems to contribute to the superior DP performance found among specialized LDS in comparison with ARS.

## Introduction

Competitive cross-country (XC) skiing consists of the Olympic disciplines, with the competition formats ranging from short sprint competitions (~1.3–1.8 km) to 30- and 50-km races performed in undulating terrain, and long-distance XC skiing (Ski Classics) consisting of distances ranging from 40 to 90 km performed in flatter terrain using the classical style. All-round skiers (ARSs) competing in the Olympic disciplines are known for their high maximal oxygen uptake (VO_2max_), as well as high technique-specific peak oxygen uptakes (VO_2peak_) and gross efficiency (GE) in the main sub-techniques of the classical and skating styles (Saltin, 1997; Sandbakk and Holmberg, 2014, 2017; Holmberg, 2015). While the Olympic XC skiing disciplines include the use of, and constant changes between, many different sub-techniques (Sandbakk and Holmberg, 2017; Strøm Solli et al., 2018, 2020), the flatter course profiles in long-distance XC skiing events have led to an extensive and, at the elite level, almost exclusive use of the double poling (DP) sub-technique (Sagelv et al., 2018; Zoppirolli et al., 2018; Skattebo et al., 2019; Stöggl et al., 2020).

To adapt to these competitive demands, specialized long-distance XC skiers (LDSs) perform a higher percentage of their total training volume using DP than ARS (~50 vs. ~25%) (Torvik et al., 2021). Due to the higher volumes of DP training among LDS, superior technique-specific physiological adaptations and greater upper-body strength and power may be expected in comparison with typical ARS. In this context, Skattebo et al. (2019) found lower oxygen cost and better GE during submaximal DP in LDS compared to ARS. In the same study, LDS achieved similar DP performance in the laboratory (peak speed and time to exhaustion) as the ARS despite obtaining lower VO_2peak_ values in both DP and running, as well as lower performance during a time to exhaustion test in running (Skattebo et al., 2019).

The higher proportion of the annual training volume performed exclusively in DP by LDS targeting the upper-body adaptations may also positively influence DP speed and efficiency by improving technical solutions (Stöggl et al., 2007) and delaying fatigue in long-distance DP races (Sandbakk, 2018). However, Skattebo et al. (2019) found no kinematic differences that could possibly explain the observed differences in submaximal oxygen cost during submaximal DP between LDS and ARS. Contrary, Zoppirolli et al. (2020) have previously shown that the best LDSs are able to maintain speed and cycle length better than their lower-performing counterparts in the long-distance race Marcialonga, although the technical solutions in DP among specialized LDS are currently understudied. Accordingly, detailed examination of the underlying mechanisms related to the engagement of different body segments and EMG amplitude of muscles in DP should be explored in LDS and compared to the patterns obtained from ARS.

The technical solutions associated with DP performance in ARS include a distinct extended hip, knee, and ankle (the “high hip, high heel” strategy), with a clear forward lean of the body during the poling phase (Holmberg et al., 2005; Lindinger et al., 2009a; Danielsen et al., 2015). This high initial position followed by rapid downward–forward body movement during propulsion through trunk, hip, knee, and ankle flexion increases the ability to rapidly generate pole force during a short and dynamic poling phase. Furthermore, Holmberg et al. (2005) and Stöggl and Holmberg (2011) emphasized the importance of a more vertical pole plant during DP in relatively flat terrain. A more vertical pole plant, apart from increasing pole contact time, is considered important for muscle pre-activation (and time to build up force) and flexion–extension elbow (and shoulder) angle patterns (Holmberg et al., 2005; Lindinger et al., 2009a,b). Based on EMG, Holmberg et al. (2005) found proximo-distal sequential muscle activation patterns in the upper body during DP, with EMG amplitude of rectus femoris and rectus abdominis preceding that of latissimus dorsi and triceps brachii. However, it is not known whether such characteristics may differ between ARS and LDS, which could indicate differences in movement timing and kinematic. Furthermore, Zoppirolli et al. (2015) found that elite skiers had a more advantageous movement of center of body mass (CoM) in DP than lower-ranked skiers, with less downward and more forward CoM movements in the poling phase. Whether skiers specialized in DP have further developed such technical solutions or use them differently than less DP specialized ARS remains to be elucidated.

Therefore, the aim of this study was to compare physiological and kinematic responses to DP, as well as upper-body strength and power between LDS and ARS. Methodologically, the groups were matched for VO_2max_ in running and overall training volume. Since LDSs perform more of their total training volume using DP, it was hypothesized that specialized LDS would achieve better DP performance, and higher VO_2peak_ and GE in DP than ARS, and that these differences would coincide with better technical solutions among LDS.

## Methods

### Participants

A number of 12 Norwegian male, competitive XC skiers, including five world-class LDS competing primarily in Ski Classics and seven ARS competing in all-round skiing, volunteered to participate in this study. Both groups displayed approximately equal characteristics in terms of overall training volume and overall aerobic capacity as measured by VO_2max_ in running (RUN-VO_2max_). The participants' ages, anthropometrics, physiological characteristics, and training volumes are presented in Table 1.

**Table 1:** Age, anthropometrics, physiological characteristics, and training volumes of five world-class long-distance skiers (LDS) and seven elite male all-round skiers (ARS).

**Variable**	**LDS (n=5)**	**ARS (n=7)**	**p, g_**s**_**
Age (year)	28.8 ± 5.1	22.3 ± 2.8	0.010, 1.72
Body height (cm)	183.1 ± 7.4	182.3 ± 5.3	0.989, 0.01
Body mass (kg)	80.2 ± 7.1	74.2 ± 5.3	0.190, 0.83
BMI	23.9 ± 1.5	22.0 ± 0.7	0.156, 0.83
HR_max_ (beats·min^−1^)	191 ± 9	194 ± 9	0.434, 0.44
Annual training volume (h)	810 ± 52	780 ± 66	

*Presented as mean ± SD*.

*LDS, long-distance skiers; ARSs, all-round skiers; VO_2max_, maximal oxygen uptake in running; HR_max_, maximal heart rate in running. p-value for independent t-test between groups, g_s_ is Hedges*.

Prior to the data collection, all participants were informed about the content of the study before giving their written consent to participate. The study was approved by the Norwegian Center for Research Data and carried out in line with the current ethical standards for human participation in scientific research of the Declaration of Helsinki.

### Procedures

The skiers performed three tests on separate days in a randomized order: day (1) treadmill running tests, day (2) treadmill DP tests, and day (3) body mass scan and an upper-body pull-down exercise to determine one repetition maximum (1RM) strength and power. The performance and physiological responses were determined during both treadmill running and DP, while three-dimensional kinematics and EMG of selected muscles were obtained during DP. Before each test, the participants arrived at the laboratory in a rested and well-hydrated state, at least 2 h postprandial and without having consumed alcohol or caffeine and performed any strenuous exercise 24 h before the tests.

#### Double Poling Tests

All DP tests were performed at a treadmill inline of 5%, simulating moderate uphill terrain in long-distance races. Initially, the participants performed a 10-min warm-up at 10 km·h^−1^ [~60–70% of maximal heart rate (HR_max_)]. Before the start of the test, reflective markers and EMG electrodes were attached to the participant's body. Thereafter, one 5-min submaximal workload was performed at 12.5 km·h^−1^ in which steady-state metabolic rates were achieved. After a 2-min break, an incremental test to exhaustion was performed to determine VO_2peak_ in DP (DP-VO_2peak_) and performance measured as peak speed (DP-V_peak_). The test started at 15 km·h^−1^ with increasing speed by 1.5 km·h^−1^ every minute until the voluntary exhaustion. Termination was defined as when the skiers could no longer keep up and their roller-ski wheels crossed a mark in the middle of the treadmill. Performance (DP-V_peak_) was defined as: V_peak_ = V_c_ + [(t_final_/60) × ΔV] with V_c_ the speed of the last completed workload, t_final_ the duration of the last workload, and ΔV the change in speed between each workload. DP-VO_2peak_ was defined as the average of the two highest consecutive VO_2_ measurements averaged over 30-s periods, and peak heart rate (HR_peak_) as the highest heart rate during a 5-s period.

#### Running Tests

All running tests were performed at a treadmill incline of 10.5%. First, the participants performed a 10-min warm-up at 8 km·h^−1^ (~60–70% of HR_max_), followed by one submaximal 5-min workload at 10 km·h^−1^, where steady-state metabolic rates were achieved. Thereafter, the participants performed an incremental test to the exhaustion to determine RUN-VO_2max_ and performance measured as V_peak_ (RUN-V_peak_). The test started at 10 km·h^−1^ with the speed subsequently increased by 1 km·h^−1^ every minute until voluntary exhaustion. RUN-VO_2max_, RUN-V_peak_, and HR_max_ were calculated in the same way as in the abovementioned DP test. We chose to perform a running test rather than a more ski-specific test with diagonal stride on roller skis, mainly because our LDS had performed very limited training using diagonal stride over the last years, which is a typical trend in the training routines of modern LDS. In contrast, they typically perform extensive amounts of running (especially high-intensity sessions), similar to the training of ARS (Skattebo et al., 2019).

#### Upper-Body Strength and Power

After a 10-min running warm-up (~60–70% of HR_max_), 1RM strength, and power were determined in a pull-down exercise simulating the DP movement emphasizing elbow extension, shoulder extension, and trunk flexion movements (Hegge et al., 2016; Østerås et al., 2016). Sitting position was adjusted at the cable pull-down apparatus to ~90° angles in the knee and ankle joints and a stable back at ~120° angle to the seat. The skiers were strapped around the hip to the seat to isolate muscle work mainly in the upper body, excluding most of the possibility to use the lower body. Before their maximal effort in 1RM strength, the skiers performed ten repetitions at 60%, eight repetitions at 70%, six repetitions at 80%, and three repetitions at 90% of their estimated 1RM based on the familiarization with the same exercise. Thereafter, 1RM was determined by increasing the load by 1.25–2.5 kg per attempt until 1RM was achieved, and the participants failed to perform the exercise correctly. There was a 2-min break between each 1RM attempt. The 1RM results were further converted to mean power by multiplying mass with mean velocity of the pull-down movement, as previously described (Vandbakk et al., 2017). Mean velocity was measured with a linear encoder at 200 Hz (Muscle Lab Power, Ergotest Innovation AS, Porsgrunn, Norway), and data were processed with the associated computer software program (MuscleLab 3010E, software version 7.17; Ergotest Technology AS).

#### Anthropometrics or Body Composition

All participants were assessed for body composition immediately upon their arrival at the laboratory on the morning of test day 3 using the InBody 770 device (InBody 770, Cerriots, CA, USA). During the test, the participants wore only underpants, while all metal, watches, and jewelry were removed, and they stood barefoot on the electrodes in the platform. The participants held their thumb and fingers in direct contact with the electrodes on the handles. They stood with their elbows extended and their shoulder joint abducted at a 30° angle for ~60 s while body composition was determined. This included body mass, fat-free mass, and the distribution of total body mass in the trunk, legs, and arms. When comparing actual fat-free mass and fat mass, the previous studies have shown that the InBody 770 is a valid alternative to dual energy X-ray absorptiometry (DEXA) in trained men and women (Antonio et al., 2019), but the device slightly overestimates fat-free mass and underestimates fat mass by ~1–4.5% (Brewer et al., 2021), depending on the age, sex, training state, food intake, and time of testing.

### Measurements and Analysis

#### Running and DP Tests

The running and DP tests were performed on a 5 m × 3 m motor-driven treadmill (Forcelink Technology, Zwolle, The Netherlands). All participants used the same pair of classic roller skis with the standard wheels of resistance category 2 (IDT Sports, Lena, Norway). They used their own poles with special carbide tips to ensure optimal grip on the treadmill. During the incremental DP test, the participants were secured with a safety harness connected to the emergency brake of the treadmill. A towing test was performed to determine the coefficient of rolling resistance (μ) of the roller skis before and after all tests. The mean value of μ was 0.018 ± 0.001.

#### Respiratory Variables

Respiratory variables were measured using open-circuit indirect calorimetry with a mixing chamber and 30-s averaging of the variables measured (Oxycon Pro, Jaeger GmbH, Hoechberg, Germany). The instruments were calibrated against ambient air conditions and certified gases of known concentrations of O_2_ (15.0 ± 0.04%) and CO_2_ (5.0 ± 0.01%) before each test session. The flow transducer (Triple V, Erick Jaeger GmbH, Hoechberg, Germany) was calibrated using a 3-L high-precision calibration syringe (Hans Rudoph Inc., Kansas City, MO, USA). Heart rate was measured with a Polar heart rate monitor (V800, Polar, Finland), whereas blood lactate concentrations were obtained from 20 μl of fingertip blood analyzed using the stationary Biosen C-Line lactate device (Biosen, EKF Industrial Electronics, Magdeburg, Germany). Rating of perceived exertion (RPE) was determined using the 6–20 Borg Scale (Borg, 1970).

#### Gross Efficiency

Gross efficiency during steady-state workloads in both running and DP was calculated by dividing work rate by metabolic rate (e.g., van Ingen Schenau and Cavanagh, 1990). Work rate was calculated as the rate of work done against gravity and rolling resistance: mgv[sin(α) + cos(a)μ], where m is body mass, g is acceleration of gravity (9.81), v is treadmill speed, and α is the angle of the treadmill. Metabolic rate was obtained by converting the average VO_2_ and RER of the final minute of the submaximal workloads and calculated according to the study of Péronnet and Massicotte (1991). In running, the rate of work done against rolling resistance is zero, and thus, work rate was mgvsin(α).

#### Kinematics

A three-dimensional motion capture system (Qualisys, Gothenburg, Sweden) consisting of eight Oqus 400 cameras captured position data of reflective markers at a frequency of 250 Hz using Qualisys Track Manager. The 3D motion capture system was synchronized with the EMG recordings, using MuscleLab 6000 (Ergotest Technology AS, Langesund, Norway). For both kinematics and EMG, at least 10 full movement cycles at the workload of each participant were obtained and used for further analysis. Reflective markers were placed on the right side of the body on the following anatomical landmarks: styloid process of ulna, lateral epicondyle of humerus, lateral end of acromion process, greater trochanter, lateral epicondyle of femur, lateral malleolus (on the ski boot), and head of fifth metatarsal (on the ski boot) (Winter, 2009). These markers defined six body segments: foot, shank, thigh, trunk (including head), arm, and forearm. One marker was placed 10 cm below the right pole grip and one marker was placed at the bottom of the right pole tip. Raw position data were low-pass filtered (fourth-order Butterworth) at 15 Hz. Segment position data were used to calculate body center of mass using de Leva (de Leva, 1996) segmental inertial properties. Joint angles (elbow, shoulder, hip, knee, and ankle) and pole angle were calculated as described by Danielsen et al. (2018). Time between pole on and off defined the poling phase, and consecutive pole plants defined one movement cycle, while the time between pole off and on defined the swing phase. The instants of pole on and off were defined using the (peak) second derivative of pole tip marker position data. Kinematics were analyzed in MATLAB (R2019b, Mathworks Inc., Natick, MA, USA).

#### Electromyography

Electromyography (EMG) was measured according to the recommendations of the SENIAM (Hermens et al., 2000), using MuscleLab system v.10.5.60 (Ergotest AS, Porsgrunn, Norway). EMG was measured in nine muscles: triceps brachii, erector spinae at L4–L5, rectus abdominis, latissimus dorsi, gluteus maximus, biceps brachii, rectus femoris, gastrocnemius, tibialis anterior, and biceps femoris. The skin was prepared by shaving, abrading, and cleaning with isopropyl alcohol to reduce skin impedance before positioning the electrodes over each muscle. To strengthen the signal, a conductive gel was applied to self-adhesive electrodes (Dri-Stick Silver circular sEMG Electrodes AE-131, NeuroDyne Medical, Cambridge, MA, USA). The electrodes (11 mm contact diameter, 20 mm center-to-center distance) were placed on the participant's right side. To minimize noise from external sources, the EMG raw signal was amplified and filtered using a preamplifier located as near to the pickup point as possible. The common-mode rejection ratio was 106 dB, and the input impedance between each electrode pair was >10^12^ Ω. The EMG signals were sampled at a rate of 1,000 Hz. Signals were band-pass filtered (fourth-order Butterworth filter) with cutoff frequencies of 20 and 500 Hz and converted to root mean square signals using a hardware circuit network to create the linear envelope of the EMG signal (frequency response 450 kHz, averaging constant 12 ms, total error ± 0.5%) (van den Tillaar and Saeterbakken, 2014).

All kinematics and EMG data were time normalized for each participant and cycle and averaged over ~10 cycles for each speed. For each muscle, the peak EMG and the timing (occurrence of peak EMG in relation to normalized cycle time) at each speed were calculated. Cycle average normalized EMG (nEMG_avg_) at all speeds was computed by normalizing cycle average EMG of each submaximal speed to peak EMG measured at V_peak_, as recommended for high-velocity dynamic movements such as sprint running (Albertus-Kajee et al., 2011; Ball and Scurr, 2013) and high-speed DP.

### Statistical Analysis

All results are presented as mean ± standard deviations unless otherwise specified, and statistics were analyzed using SPSS version 27.0 (IBM Corp., Armonk, New York, USA) and Microsoft Excel 2017. Because all the participants did not complete all speeds, most statistical analysis of kinematics and EMG was restricted to speeds between 12.5 and 21.0 km·h^−1^. Statistical analysis was performed using linear mixed models (LMM, with restricted maximum likelihood estimation), using the mixed command in SPSS, with participant-specific intercepts. To compare _n_EMG_avg_ and timing of peak EMG between groups, a LMM was applied with group (LDS, ARS) and speed (12.5–21.0 km·h^−1^) as the fixed factors. To compare the timing of peak EMG between the nine muscles, an LMM was applied with group and muscle (nine muscles) as fixed factors. A LMM was used to compare the effects of group and speed (12.5–21.0 km·h^−1^), as well as their interaction effects, on kinematics. Between-group comparisons of variables at submaximal and peak workloads, as well as possible differences in strength, power, and body composition, were compared using independent Welch's *t*-tests (Delacre et al., 2017). Despite small number of participants, variables were approximately normally distributed in each group (variables and residuals assessed by normal QQ plots). Effect sizes for local differences were calculated as Hedges' g_s_ (g_s_) (Lakens, 2013), where 0.2–0.5 constitutes a small effect, 0.5–0.8 a medium effect, and >0.8 a large effect (Cohen, 1988).

## Results

### Body Composition and 1RM Upper-Body Strength

Table 2 shows body composition measures and upper-body strength and power for both LDS and ARS. The LDSs were heavier than ARS, and LDS also had more muscle mass located in the upper body and arms but a lower percentage of muscle mass in the legs. The LDS tended to display higher 1RM in the pull-down exercise than ARS, with no difference in mean power found between the two groups.

**Table 2:** Body composition and 1RM upper-body strength and power for all participants pooled, five world-class long-distance skiers (LDS) and seven elite male all-round skiers (ARS).

**Variables**	**Pooled (n=12)**	**LDS (n=5)**	**ARS (n=7)**	**p, g_**s**_**
**Body composition**				
Total mass (kg)	76.7 ± 4.5	80.2 ± 7.1	74.2 ± 5.3	0.190, 0.83
Muscle mass (kg)	40.2 ± 3.7	41.8 ± 4.3	39.1 ± 2.7	0.259, 0.72
Body mass index	23.1 ± 1,5	23.8 ± 0.6	22.5 ± 1.7	0.115, 0.83
Upper body (kg)	30.6 ± 2.7	32.0 ± 3.2	29.6 ± 1.8	0.219, 0.83
Percentage of total mass (%)	39.9 ± 1.7	39.9 ± 0.9	40.0 ± 2.0	0.919, 0.05
Arms (kg)	8.1 ± 0.9	8.6 ± 1.1	7.8 ± 0.6	0.186, 0.89
Percentage of total mass (%)	10.6 ± 0.6	10.7 ± 0.5	10.5 ± 0.7	0.469, 0.39
Legs (kg)	21.4 ± 1.9	21.7 ± 2.4	21.1 ± 1,4	0.664, 0.27
Percentage of total mass (%)	27.9 ± 1.2	27.1 ± 0.9	28.5 ± 1.0	0.037, 1.31
**Upper-body strength**
1RM (kg)	117.7 ± 9.4	121.5 ± 6.5	113.9 ± 12.2	0.198, 0.68
1RM/total mass (kg)	1.5 ± 0.1	1.5 ± 0.1	1.5 ± 0.2	0.851, 0.10
Power (W)	748.4 ± 101.0	763.6 ± 67.6	733.1 ± 134.3	0.618, 0.25
Power/total mass (W/kg)	9.7 ± 1.3	9.9 ± 0.8	9.5 ± 1.8	0.776, 0.16

*Presented as mean ± SD*.

*1RM, one repetition maximum. p-value for independent t-test between groups, g_s_ is Hedges*.

### Performance and Physiological Responses

The LDS achieved higher DP-V_peak_ than ARS, although no difference in RUN-V_peak_ was found between groups (Table 3). Absolute values in RUN-VO_2max_ were higher in LDS than in ARS, although no between-group differences were found for body mass normalized RUN-VO_2max_ (Table 3). Both absolute and body mass normalized DP-VO_2peak_ were higher for the LDS compared to the ARS. Thus, the DP-VO_2peak_ to RUN-VO_2max_ ratio was higher in the LDS than in ARS (97 vs. 94%, *p* = 0.075, g_s_ = 1.08). A similar pattern was found for HR_peak_, with the LDS reaching 98% of their RUN-HR_max_ in DP compared to 95% in ARS (*p* = 0.154, g_s_ = 0.83). During submaximal DP, LDS displayed lower oxygen cost and higher GE than ARS (Table 3), with similar patterns observed for submaximal running, although the group differences in running were smaller.

**Table 3:** Performance and physiological responses to submaximal and incremental DP (at 5% incline) and running (at 10.5% incline) in five world-class long-distance skiers (LDS) and seven elite male all-round skiers (ARS).

**Variables**	**Double poling**			**Running**		
	**LDS**	**ARS**	**p, g_**s**_**	**LDS**	**ARS**	**p, g_**s**_**
**Performance test**
V_peak_ (km·h^−1^)	22.1 ± 1.0	20.7 ± 0.9	0.040, 1.36	15.1 ± 1.0	14.6 ± 0.8	0.475, 0.42
Peak work rate (W)	336 ± 39	289 ± 33	0.060, 1.22	350 ± 39	320 ± 21	0.176, 0.93
VO_2max/peak_ (mL·min^−1^·kg^−1^)	68.3 ± 2.1	65.1 ± 2.7	0.043, 1.20	70.5 ± 2.8	69.1 ± 4.2	0.524, 0.33
VO_2max/peak_ (L·min^−1^)	5.5 ± 0.6	4.8 ± 0.3	0.083, 1.31	5.6 ± 0.5	5.1 ± 0.3	0.094, 1.19
RER (–)	1.10 ± 0.03	1.12 ± 0.06	0.467, 0.37	1.17 ± 0.02	1.15 ± 0.05	0.366, 0.45
HR_max/peak_ (bpm)	187 ± 4	184 ± 10	0.466, 0.36	191 ± 4	194 ± 8	0.390, 0.44
Borg (6–20)	18.2 ± 1.3	19.0 ± 1.2	0.304, 0.61	19.6 ± 0.5	19.3 ± 0.5	0.335, 0.57
**Submaximal test**
Speed (km·h^−1^)	12.5	12.5		10	10	
VO_2_ (mL·min^−1^·kg^−1^)	39.4 ± 1.2	42.2 ± 2.6	0.033, 1.21	52.8 ± 0.8	54.6 ± 2.2	0.090, 0.91
VO_2_ (L·min^−1^)	3.15 ± 0.32	3.14 ± 0.30	0.960, 0.03	4.22 ± 0.42	4.06 ± 0.39	0.530, 0.39
RER (–)	0.88 ± 0.04	0.94 ± 0.04	0.029, 1.45	0.94 ± 0.02	0.94 ± 0.04	0.875, 0.08
Metabolic rate (W)	1074 ± 102	1087 ± 110	0.829, 0.12	1466 ± 170	1408 ± 106	0.524, 0.40
Work rate (W)	185 ± 19	172 ± 13	0.247, 0.74	217 ± 23	202 ± 15	0.247, 0.74
Gross efficiency (%)	17.2 ± 0.5	15.9 ± 1.1	0.019, 1.38	14.8 ± 0.2	14.3 ± 0.5	0.069, 1.00

*Presented as mean ± SD. p-values and g_s_ are reported for group comparisons (independent t-tests)*.

*VO_2max_, maximal oxygen uptake; VO_2peak_, peak oxygen uptake; RER, respiratory exchange ratio; HR_max_, maximal heart rate; HR_peak_, peak heart rate; VO_2_, oxygen uptake. p-value for independent t-test between groups, g_s_ is Hedges*.

### Kinematics

**Figure 1:**
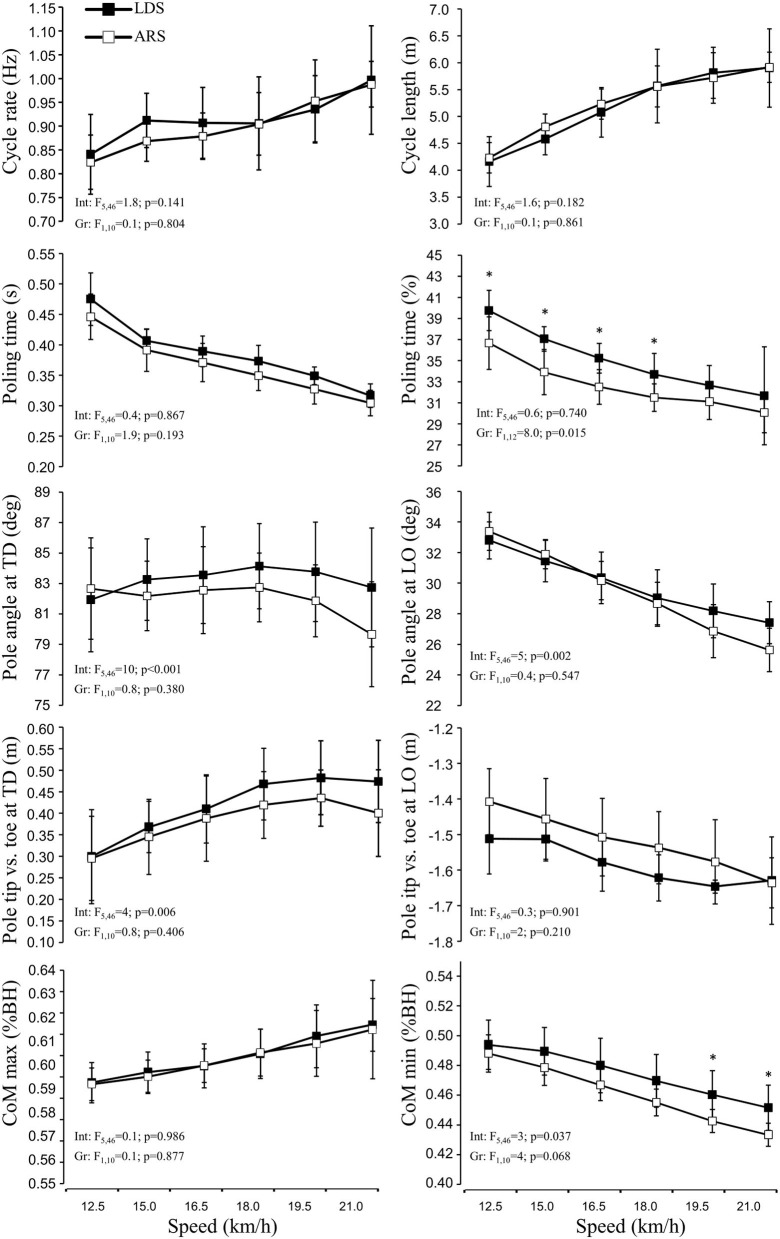
Kinematic responses as a function of speed in long-distance (LDS, *n* = 5) vs. all-round (ARS, *n* = 7) cross-country skiers performing treadmill DP at 5% inclination. Int, interaction; Gr, group. *Indicates *p* <0.05.

There were no between-group differences in cycle length or cycle rate at any speed (Figure 1), with no differences in peak values for rate (1.12 ± 0.18 vs. 1.04 ± 0.08 Hz, *p* = 0.385, g_s_ = 0.58) or length (6.0 ± 0.7 vs. 5.7 ± 0.6 m, *p* = 0.503, g_s_ = 0.39). The LDS had slightly longer poling times (*p* = 0.193) than ARS, while relative poling times differed (*p* = 0.015) at speeds between 12.5 and 18.0 km·h^−1^ (40–34 vs. 37–31% in LDS and ARS, respectively). Significant interaction effects were found for pole angle at touchdown and lift-off (more vertical for LDS), and for the distance between pole tip and feet at touchdown (longer distance for LDS), and the LDS seemed better able to maintain these characteristics at higher speeds (Figure 1).

**Figure 2:**
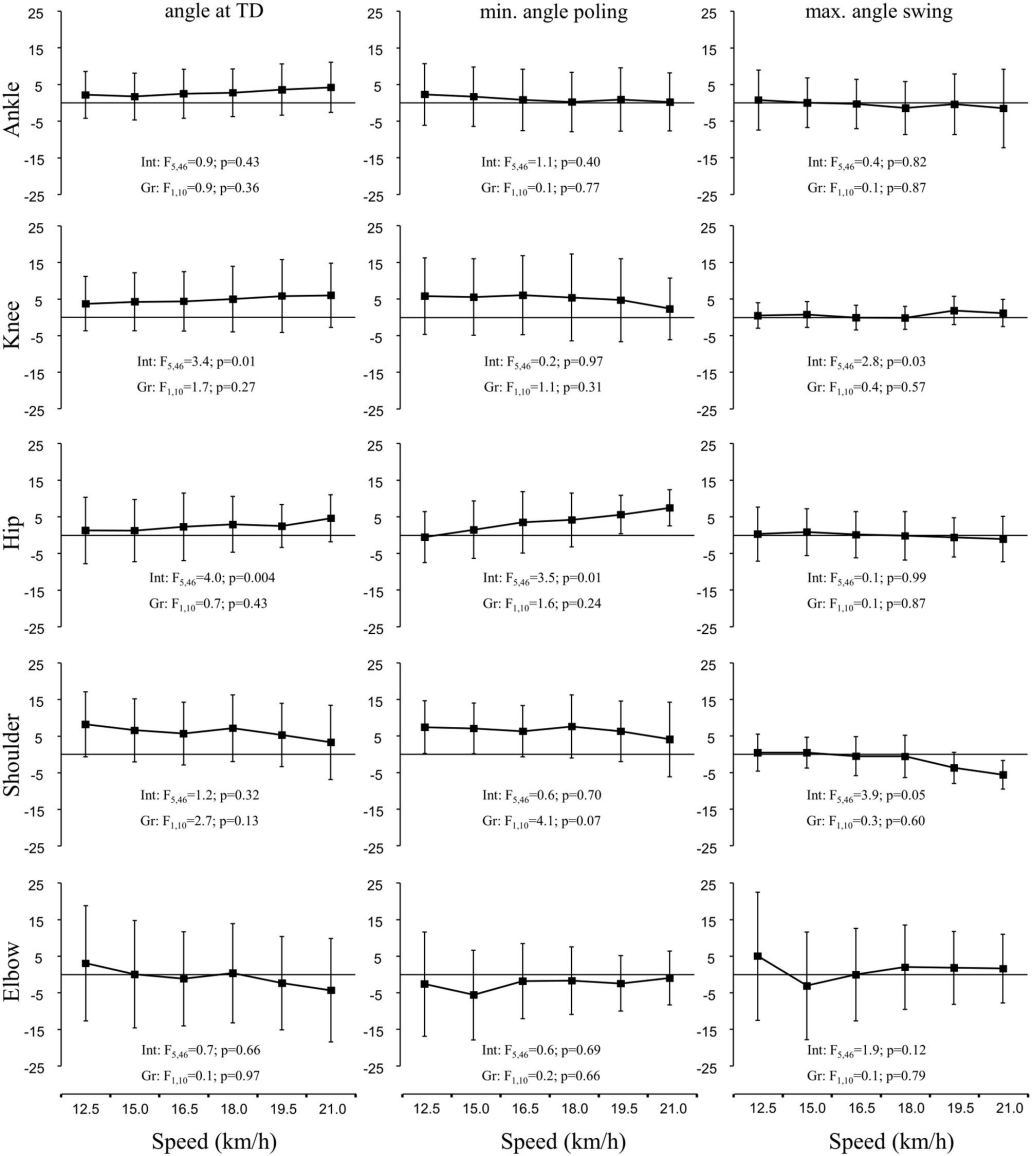
The mean group difference (long-distance minus all-round cross-country skiers) and 95% CI of joint angles at touch down **(left panels)**, the minimum angle reached within the poling phase (maximum angle for the shoulder) **(middle panels)**, and maximum angles reached within the swing phase **(right panels)** during treadmill DP at 5% inclination. Int, interaction; Gr, group.

Although joint angles appeared to be very similar in both groups (Figure 2), interaction effects were found for knee and hip angles at touchdown and for minimum hip angle during the poling phase, with the LDS maintaining a slightly more extended hip positioning at faster speeds (Figure 2). These differences led to the significant interaction and minor group effects on the minimum center of mass height.

### EMG

**Figure 3:**
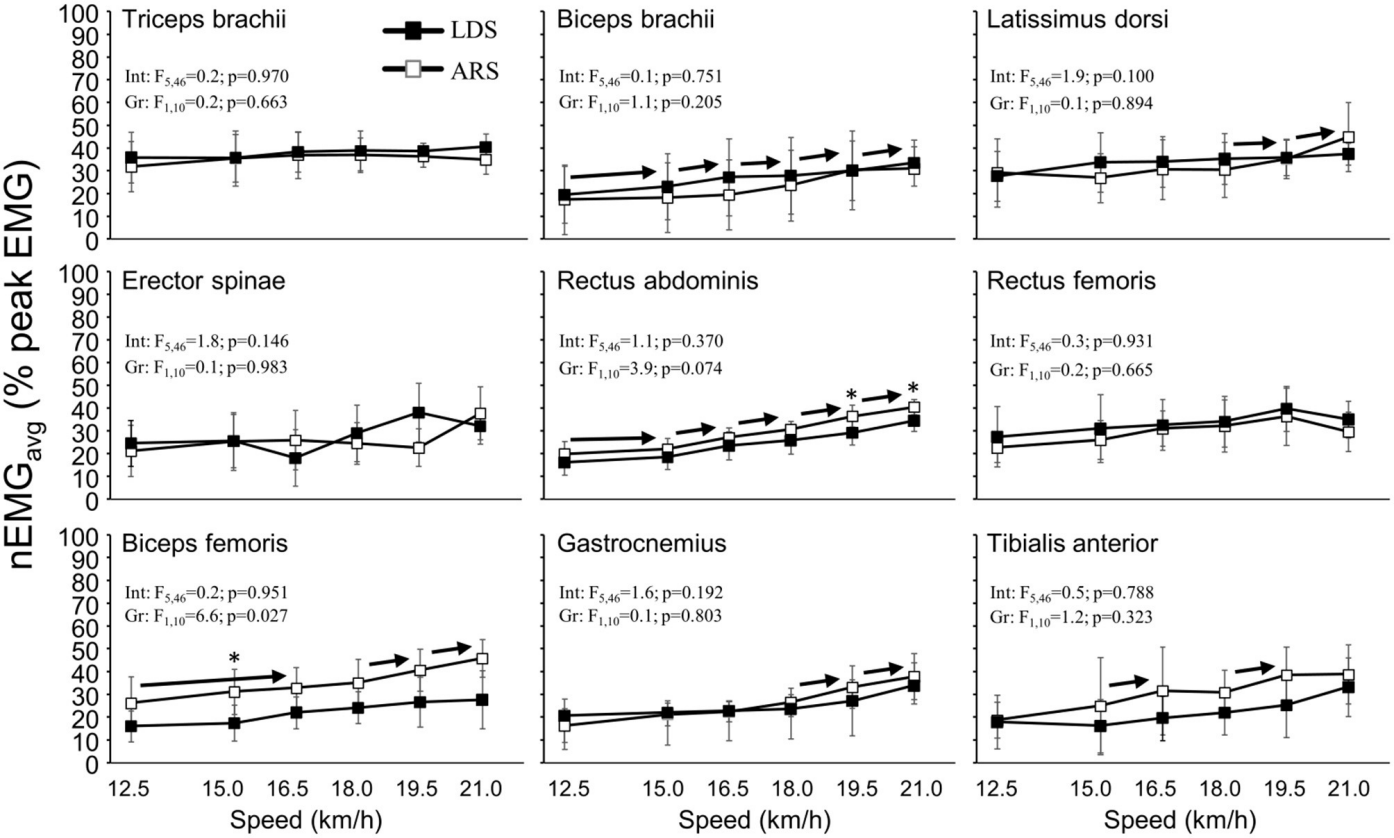
Average normalized EMG (nEMG_avg_) in long-distance (LDS, *n* = 5) vs. all-round (ARS, *n* = 7) cross-country skiers performing treadmill DP at 5% inclination. Int, interaction; Gr, group. *Indicates *p* < 0.05. → Indicates a significant increase in EMG amplitude between these velocities and all right of the sign (*p* < 0.05).

Double poling speed affected nEMG_avg_ of most muscles (*p* < 0.001, Figure 3) but less so for triceps brachii (*p* = 0.202), erector spinae (*p* = 0.177), and rectus femoris (*p* = 0.620). nEMG_avg_ showed a particular increase at speeds above 18 km·h^−1^. No clear group vs. speed interaction effects for any muscles were found (Figure 3). However, nEMG_avg_ in rectus abdominis (*p* = 0.074) and biceps femoris (*p* = 0.027) were consistently slightly higher in the ARS than in the LDS (Figure 3). For some muscles, a large standard deviation (SD) reflect somewhat lower or inconsistent EMG amplitudes between skiers.

**Figure 4:**
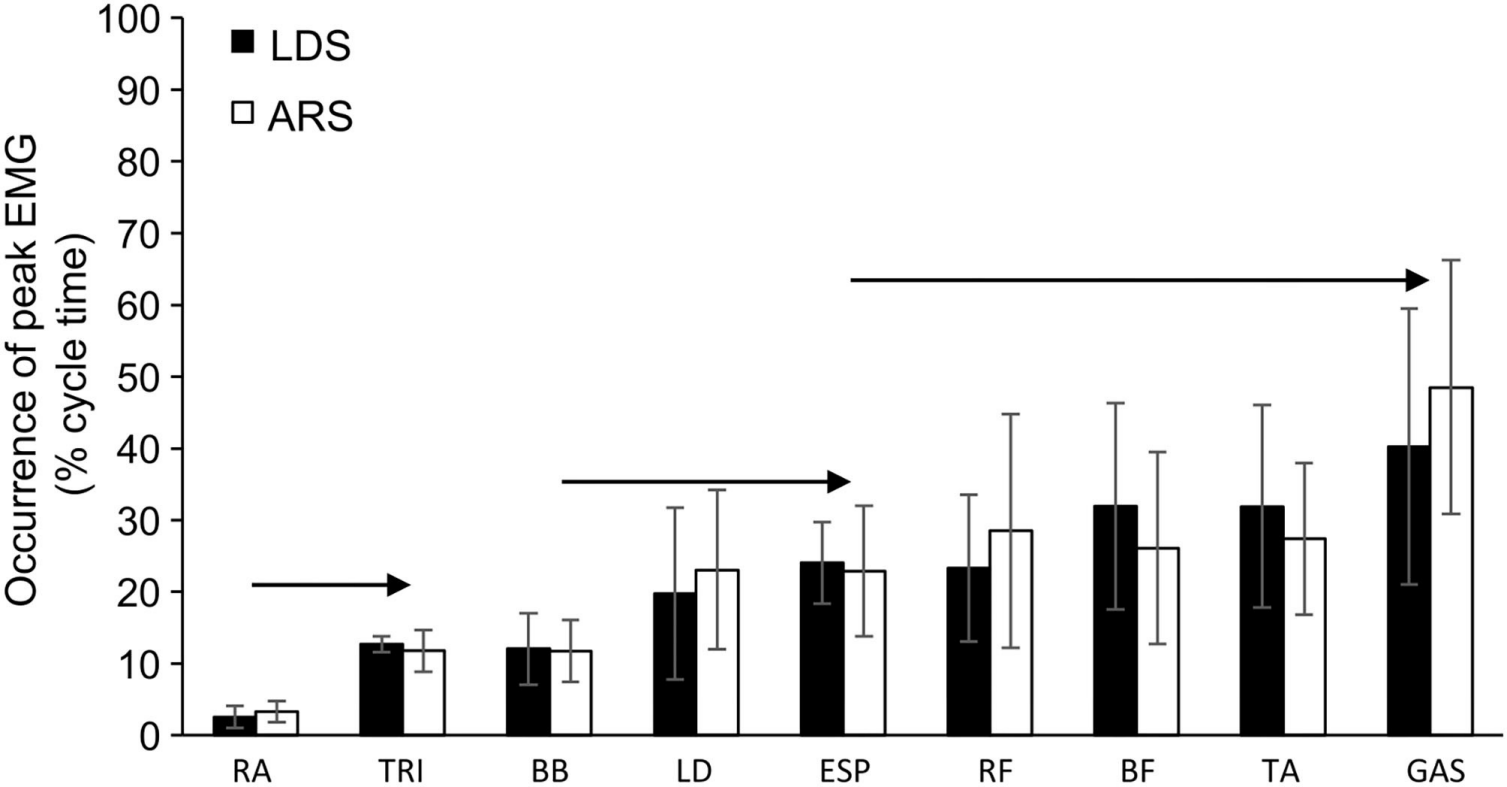
Occurrence of peak EMG amplitude during the cycle (average across all speeds) for all round (ARS, *n* = 7) and long distance (LDS, *n* = 5) cross-country skiers performing treadmill DP at 5% inclination. RA, rectus abdominis; TRI, triceps brachii; BB, biceps brachii; LD, latissimus dorsi; ESP, erector spinae; RF, rectus abdominis; BF, biceps femoris; TA, tibialis anterior; GAS, gastrocnemius. → Indicates a significant different to the muscles to the right of the sign (*p* < 0.05).

Peak EMG amplitude occurred slightly earlier at faster speeds for triceps brachii, latissimus dorsi, and rectus abdominus (*p* < 0.01), with no difference between groups for any muscle at any speed. When analyzing the timing of peak EMG amplitude for all muscles across all speeds, an effect of muscle was found (*p* < 0.001), without any group (*p* = 0.520) or interaction effects (*p* = 0.841; Figure 4).

## Discussion

The aim of this study was to compare physiological and kinematic responses to DP, as well as upper-body strength and power between LDS and ARS. The main findings were that LDS achieved better DP performance than ARS, which were coincided by higher DP-VO_2peak_, DP-VO_2peak_/RUN-VO_2max_ ratio, and GE. In addition, our data indicated that LDS maintained more effective technical patterns at higher speeds, as indicated by the significant interaction effects for pole angle at touchdown and maximum knee and hip flexion angles during the poling phase, implying that LDS maintained a more upright body position with more vertically angled poles throughout the poling phase. Finally, nEMG_avg_ in rectus abdominus were higher in ARS than in LDS, with a similar pattern indicated for biceps femoris.

### Physiological Responses

As expected from their large amounts of DP-specific training (Torvik et al., 2021), LDS reached higher DP-V_peak_ in comparison with ARS during the incremental test to exhaustion with no between-group differences observed in RUN-V_peak_. Better DP performance in LDS was coincided by higher DP-VO_2peak_, DP-VO_2peak_/RUN-VO_2max_ ratios (i.e., 97% in LDS vs. 94% in ARS), as well as with higher GE during submaximal DP. These findings differ partly from the study of Skattebo et al. (2019), who found no difference between LDS and ARS in DP performance using a comparable design. These conflicting findings are most likely explained by different study groups, number of participants included, and the matching of LDS vs. ARS. Skattebo et al. (2019) matched the groups for overall performance level (elite ARS vs. elite LDS), whereas the groups of this study were matched for RUN-VO_2max_ and overall training volume. In Skattebo et al. (2019), ARS achieved both higher RUN-VO_2max_ and DP-VO_2peak_ compared to LDS, although with similar DP-VO_2peak_/RUN-VO_2max_ ratios observed between groups. Despite this, DP performance of LDS was identical to that of the ARS. Thus, independent of the different matching between groups, both the data of Skattebo et al. (2019) and this study therefore indicate that large amounts of DP-specific training lead to improved DP performance, beyond what may be expected based only on, for example, DP-VO_2peak_.

The ability to reach high VO_2peak_ values is generally dependent on exercise modality, and the ability to generate high power within that particular modality. Therefore, the amount of muscle mass engaged in generating power is important (Noakes, 2004). The fact that XC skiers can reach more than 90% of their RUN-VO_2max_ during DP further demonstrates that DP involves whole-body work (Van Hall et al., 2003; Holmberg et al., 2006; Hegge et al., 2016; Danielsen et al., 2018). The difficulty of reaching VO_2max_ in DP is likely to be related to longer diffusional distances, shorter mean transit times, and lower oxidative capacity in the upper than the lower body (Van Hall et al., 2003; Calbet et al., 2005; Ortenblad et al., 2018). Therefore, upper-body muscles are reported to extract ~10% lower O_2_ than leg muscles (Calbet et al., 2005) and contribute, together with a lower vascular conductance (Calbet et al., 2004), to lower VO_2peak_ values in DP compared to running (Losnegard et al., 2019). Recently, Berg et al. (2019) found higher mitochondrial respiration in the upper body but equal in the lower body when comparing XC skiers and physically active controls. It may be hypothesized that the average DP-VO_2peak_/RUN-VO_2max_ ratio among LDS in this study, which to our knowledge is the highest ever reported in the literature (Losnegard, 2019), is due to the high volumes of DP-specific training in LDS which may further increase O_2_ extraction and/or enhance mitochondrial respiration in upper-body muscles beyond what has previously been shown. In support of this, we additionally found small differences between LDS and ARS in body composition, with the LDS having more muscle volume in the upper body and arms. Accordingly, the more DP-specific training among LDS may induce better aerobic energy delivery, allowing LDS to reach a higher DP-VO_2peak_ and thereby achieve higher DP performance.

The higher GE and lower oxygen cost in LDS than ARS during submaximal DP in this study agrees in part with the findings of Skattebo et al. (2019), who found lower oxygen cost in LDS than ARS, but smaller differences in GE (17.2% for ARS vs. 17.9% for LDS at 252 W). This discrepancy in GE can partly be explained by the higher work rate of LDS compared to ARS in our data, due to their higher body mass because of the non-zero offset of the metabolic–work rate relationship (Ettema and Lorås, 2009; Curran-Everett, 2013). Our findings on oxygen cost and GE during submaximal running further illustrate this point, with these values also being slightly better for LDS than ARS. However, the group differences are larger for DP than for running, demonstrating an effect beyond what can be explained by work rate. Also, although oxygen cost (values relative to body mass) suffers from the same problem with ratios, these values are more interpretable as body mass is transported against gravity in our protocol as well as during XC skiing races. The speculation above concerning higher mitochondrial respiration in the upper-body muscles of LDS compared to ARS, due to more DP-specific training over more years, may also be a possible explanation for the higher GE of LDS. In any case, despite more upper-body and arm muscle mass (and total mass)—forcing LDS to generate higher work rates at a given speed than ARS—LDSs do so at equal or lower metabolic rates than ARS. Therefore, both our data and those of Skattebo et al. (2019) on oxygen cost and GE during submaximal DP suggest that skiing efficiency or economy is coupled to DP performance. In short performance tests, but probably more so in long-distance races, efficiency and economy may be more performance determinant than maximal aerobic energy delivery (Skattebo et al., 2019).

### Kinematic Responses

Due to the large amount of DP-specific training in LDS in Torvik et al. (2021), we hypothesized that better GE and performance in DP among LDS would coincide with better technical solutions. However, we found no differences in cycle length or rate between groups, either at submaximal or at high speeds to support this hypothesis. These findings are in agreement with those of Skattebo et al. (2019) and can therefore not explain the observed differences in GE and oxygen cost between groups. Although most kinematic variables were similar between groups, LDS had a longer relative poling time at most speeds, while group vs. speed interaction effects were found for pole angle (LDS more vertical poles), minimum height of the CoM within the poling phase (LDS less deep), and hip and knee angles, with all these differences previously linked to DP performance (Stöggl and Holmberg, 2011; Zoppirolli et al., 2015). The interactions found in this study therefore imply that the LDSs were better able to maintain certain technical aspects as speed increased. Moreover, nEMG_avg_ in rectus abdominus tended to be higher in ARS than in LDS, with a similar pattern indicated for biceps femoris. Thus, it seems that a range of small technique differences may help LDS to achieve higher DP-V_peak_ and better GE compared to ARS.

At all speeds, the LDS displayed slightly longer relative poling times than the ARS, but with only a minimal differences in time allowed to generate force (+0.02 s for LDS, *p* = ~0.200, g_s_ = ~0.7), which could reduce the percentage of 1RM needed to perform the DP motion (Lindinger et al., 2009b). Furthermore, more vertically planted poles have been described as a part of a preferred strategy of elite skiers to achieve a more dynamic and explosive poling phase, in which body mass is used effectively to generate pole force (Holmberg et al., 2005; Lindinger et al., 2009b; Danielsen et al., 2019, 2021). As speed increased, we found that the LDSs were able to maintain pole angle at touchdown at ~83°, while for ARS, this angle decreased toward 79–80°. The related distance between the pole tip and toe at touchdown increased from ~30 to ~48 cm from 12.5 to 19.5 km·h^−1^ in the LDS, and they were able to maintain this distance up to V_peak_, while the ARS increased this distance up to ~44 cm at 19.5 km·h^−1^ but then, it dropped at V_peak_. At higher speeds, the ability to place the poles in such advantageous positions seems to become important and may be a limitation for ARS because of the inverse relationship between muscle contraction velocity and force (Hill, 1997; Lindinger et al., 2009a).

Double poling technique in terms of joint angles also appeared very similar in both groups (Figures 2, 3). However, group vs. speed interaction effects were found for knee and hip angles at touchdown and for minimum hip angle during the poling phase, which led to the significant interaction and minor group effect on minimum CoM height (Figure 1). Although the high hip high heel DP strategy and thus considerable heightening and lowering of the CoM are the characteristics of the dynamics in modern DP (Holmberg et al., 2005; Danielsen et al., 2021), it must be performed effectively, so that the body mass (gravity) and active use of trunk flexion muscles (e.g., rectus abdominis) can be used to increase pole forces. At the same time, this strategy seems to require a certain amount of CoM lowering, and the finding that the LDS appeared to lower their CoM less than the ARS agrees with the previous findings of Zoppirolli et al. (2020) who found that the amount of CoM lowering was dependent on the skier's performance level. This might be related to keeping the amount of work required to heighten and reposition the body at a minimum (Zoppirolli et al., 2020; Danielsen et al., 2021). Overall, these findings suggest that the LDSs were able to maintain a slightly more upright body position throughout the cycle, which may explain their lower rectus abdominis nEMG_avg_ at most speeds. If the skier is not able to maintain a rather upright body position throughout the cycle, whereby the poles are planted more vertically, a greater demand might be placed on trunk flexion muscles. However, these underlying mechanisms remain speculative and must be investigated further in the future studies.

Overall, both EMG amplitude and timing of peak EMG amplitude were very similar between groups. Increasing speed led to a larger increase in nEMG_avg_ in the core and lower extremities than in triceps brachii and latissimus dorsi, which agrees with the previous findings of on-snow DP (Zoppirolli et al., 2017). The observed difference in rectus abdominis and biceps femoris nEMG_avg_ between LDS and ARS, with LDS showing lower nEMG_avg_, may further indicate that ARS work at a higher relative effort at submaximal speeds. This higher nEMG_avg_ for ARS at submaximal DP speeds may, however, be entirely due to LDS reaching higher peak speeds, with a correlation between EMG amplitudes and DP speed for these muscles. Because of this, we also normalized the RMS EMG to 12.5 km/h. This removed the group differences completely also for rectus abdominis and biceps femoris, indicating that the observed differences were due to the differences in V_peak_. Given the kinematic group differences that appear while approaching V_peak_, it can be speculated whether the lower peak EMG amplitude in ARS is explained by lower DP-V_peak_ or whether lower technical ability (including muscle coordination and neuromuscular muscle-power factors) contributes to the lower V_peak_. These statements are not mutually exclusive, and this issue should be examined further. Combined, the kinematic, strength (as well as muscle mass distribution), and EMG data of this study suggest that several factors contribute together to the observed group difference in DP-V_peak_. Working at lower relative efforts (especially in terms of oxygen cost) at a given speed will certainly contribute to delaying fatigue during long-distance events. Here, it should also be noted that we found similar sequential EMG activation patterns throughout the DP cycle as previously described by Holmberg et al. (2005), but no group differences related to timing of EMG amplitude were found in our data.

## Conclusions

This study found superior DP performance in specialized long-distance skiers compared to ARSs which coincided with higher GE and lower oxygen cost during submaximal DP combined with higher DP-VO_2peak_, as well as the highest DP-VO_2peak_/RUN-VO_2max_ ratios ever reported in the literature. Specialized LDSs also demonstrated longer relative poling times and lower normalized EMG amplitude in rectus abdominis and biceps femoris, as well as more muscle mass located in the upper body which coincided with better 1RM upper-body strength performance. In addition, specialized long-distance skiers were able to better maintain technique (i.e., more upright body position and more vertical pole angles) at faster speeds than ARSs. Taken together, the combination of better DP-specific aerobic energy delivery capacity, efficiency, and technical solutions seems to contribute to the superior DP performance found among specialized LDSs in comparison with ARSs.

## Data Availability Statement

The raw data supporting the conclusions of this article will be made available by the authors, without undue reservation.

## Ethics Statement

Ethical review and approval was not required for the study on human participants in accordance with the local legislation and institutional requirements. The patients/participants provided their written informed consent to participate in this study.

## Author Contributions

P-ØT, ØS, RT, and JD planned and designed the study. P-ØT, RT, and JD performed the data collection. P-ØT, ØS, RT, RKT, and JD analyzed and presented the data, authored, and finalized the manuscript for publication. All authors have approved the final manuscript.

## Funding

NeXt Move was funded by the Faculty of Medicine at NTNU and Central Norway Regional Health Authority. The funders had no role in study design, data collection and analysis, decision to publish, or preparation of the manuscript. The laboratory facilities and equipment were provided by NeXt Move, Norwegian University of Science and Technology (NTNU).

## Conflict of Interest

The authors declare that the research was conducted in the absence of any commercial or financial relationships that could be construed as a potential conflict of interest.

## Publisher's Note

All claims expressed in this article are solely those of the authors and do not necessarily represent those of their affiliated organizations, or those of the publisher, the editors and the reviewers. Any product that may be evaluated in this article, or claim that may be made by its manufacturer, is not guaranteed or endorsed by the publisher.

## Abbreviations

DP: double poling
RUN: running
DP-VO_2peak_: double poling peak oxygen uptake
RUN-VO_2max_: running maximal oxygen uptake
LDS: long-distance cross-country skiers
ARS: all-round cross-country skiers
XC: cross-country
DP-VO_2peak_/RUN-VO_2max_: ratio between double poling peak oxygen uptake and running maximal oxygen uptake
EMG: electromyography
nEMG_avg_: cycle average normalized EMG amplitude
GE: gross efficiency.
